# Lessons in Gynecology

**Published:** 1887-12

**Authors:** 


**Lessons in Gynecology.** By WILLIAM GOODELL, A. M., M. D., Professor of Clinical Gynecology in the University of Pennsylvania. Third edition; thoroughly revised and greatly enlarged, with 112 illustrations. Philadelphia: D. G. Brenton, 115 South Seventh street. 1887.

This edition of a most excellent work is in every way better than the preceding ones, and that is saying a great deal, for "Goodell's Gynecology" has deserved the reputation it has enjoyed. It contains the wise instruction of a thoughtful teacher, original and independent in his views, but recognized as an indefatigable worker, and the experience and knowledge gained since the last edition of the book appeared, has been drawn upon, so that every chapter has been revised, and much new matter added. The work is one which we would heartily commend to any of our readers who have not got it.

